# Editorial Department

**Published:** 1856-02

**Authors:** 


The Medical Counsellor, a spirited weekly published at Columbus, Ohio, closes its first volume with a beautifully executed steel engraving of the late Daniel Drake. We are glad to notice this evidence of prosperity on the part of our exchange. *Ceteris paribus*, an editor who has a direct pecuniary interest in the success of his journal, will make it a better one than he who has no personal responsibilities.

The New York Journal of Medicine has also a fine portrait of the late T Romeyn Beck.

